# Physical fitness and hormonal responses to strength training in adolescent elite female soccer players

**DOI:** 10.1186/s13102-026-01855-x

**Published:** 2026-07-06

**Authors:** Manel Darragi, Amira B. M. Zouita, Mariem Bousselmi, Asma Kerir, Houssem M. Karamti, Ayoub Saeidi, Anthony C. Hackney, Urs Granacher, Hassane Zouhal

**Affiliations:** 1 Higher Institute of Sport and Physical Education of SFAX, University of Sfax, Sfax, 3027 Tunisia; 2 Research Laboratory (LR23JS01) “Sport Performance, Health & Society”, Higher Institute of Sport and Physical Education of Ksar Said, Tunis, 1000 Tunisia; 3 Higher Institute of Sport and Physical Education of Tunis, University of Manouba, Tunis, 1000 Tunisia; 4 Clinical Immunology Department, Pasteur Institute of Tunis, Tunis, 1000 Tunisia; 5 Laboratory of Transmission, Control and Immunobiology of Infections (LR11IPT02), Pasteur Institute of Tunis, Tunis, 1000 Tunisia; 6 Department of Physical Education and Sport Sciences, Faculty of Humanities and Social Sciences, University of Kurdistan, Sanandaj, Kurdistan Iran; 7 Department of Exercise & Sport Science, and Department of Nutrition, University of North Carolina, Chapel Hill, NC USA; 8 Department of Sport and Sport Science, Exercise and Human Movement Science, University of Freiburg, Freiburg, Germany; 9 International Institute of Sport Sciences (2I2S), Rennes, 3500 France

**Keywords:** Resistance training, Football, Endocrine responses, Athlete monitoring, Women, Cortisol, Testosterone, GH, IGF-1

## Abstract

**Background:**

It is well-established that strength training (ST) improves physical fitness in young athletes. However, its specific effects on hormonal changes in female soccer players are unresolved.

**Objectives:**

We examined the influence of a twelve-week ST program conducted twice per week on soccer-related measures of physical fitness and hormonal profiles in adolescent elite female soccer players.

**Methods:**

Twenty-four elite young female soccer players aged 15.3 ± 0.9 years (body-mass-index [BMI]: 20.8 ± 1.9 kg/m²) and Tier 4 training and performance caliber participated in this study. Players were randomly assigned to either an ST group (STG, *n* = 12) or an active control group (CG, *n* = 12). Several parameters were determined pre- and post-training including absolute and relative to kg body mass 1-RM in bench/leg press, countermovement jump (CMJ), linear sprint speed (5-m, 10-m, 30-m sprints), speed of changing directions (T-test with and without ball), sport-specific efficiency (Yo-Yo Intermittent Level1 [YYIRTL1]) and repeated shuttle sprint ability (RSSA). Cortisol (C), testosterone (T), growth hormone (GH), and insulin-like growth factor 1 (IGF-1) were assessed using blood samples collected after 12 h of fasting, before and after the ST program.

**Results:**

No significant baseline between-group differences were observed. Statistically significant group-by-time interactions were found for muscle strength measures (e.g., relative 1-RM leg press, *p* = 0.046, d = 0.59), vertical jump performance (e.g., CMJ, *p* = 0.007, d = 1.28), and soccer-specific performance (e.g., RSSA_Mean_, *p* < 0.001, d = 2.67). Post-hoc tests demonstrated significant increases for 1-RM leg press (*p* = 0.046, d = 0.59), CMJ (*p* = 0.007, d = 1.28), and RSSA_Mean_ (*p* < 0.001, d = 2.67) in STG. Furthermore, no significant group-by-time interactions were observed for hormonal markers (T, C, GH, and IGF-1; *p* > 0.05).

**Conclusion:**

While the ST program significantly improved physical fitness in adolescent female Tier 4 soccer players, no substantial changes were observed in hormonal markers. These findings suggest that ST-related physical adaptations observed in this population may not be directly linked to the hormonal markers measured in this study.

**Trial registration:**

Clinical trial number PACTR202504552619186

https://pactr.samrc.ac.za/Search.aspx

Date of registration: 14/04/2025.

“Retrospectively registered”

Our study adheres to CONSORT guidelines.

**Supplementary Information:**

The online version contains supplementary material available at 10.1186/s13102-026-01855-x.

## Introduction

Over the last three decades, female participation in physical activity and elite sports, especially soccer, has grown significantly due to increased investment in female professional sports [1]. This growth underscores the necessity for coaches and support staff to safeguard players’ well-being and enhance performance through a comprehensive understanding of the sport’s physical demands [2]. In this context, soccer is an intermittent sport characterized by repeated high-intensity actions (sprinting, jumping, striking, accelerating, decelerating, and quick changes-of-direction [CoD]) [3], interspersed with brief recovery periods [4]. These sport-specific needs require adequate and sufficient physical fitness levels, which need to be developed in young athletes for successful performance on an elite level [5].

While the physiological demands are broadly comparable between male and female soccer players, competitive female matches typically involve a lower total distance covered (approximately 33% less than in male soccer), yet are performed at relatively higher intensities, with peak running speeds frequently exceeding 15 km/h [6]. To this end, it is essential to utilize specific training modalities, such as strength training (ST), plyometric exercises, sprint training, as well as technical and tactical drills, to comprehensively prepare young female soccer players for the physical and cognitive demands of the game [7]. Interestingly, ST has been shown to be an effective method for improving physical performance in young female soccer players, enhancing qualities such as strength, power, and overall athletic development [7–9].

In this context, for athletes inexperienced with strength and conditioning, ST guidelines recommend beginning with slower, more controlled movements to develop fundamental strength safely and ensure proper exercise technique [10]. This conservative strategy is crucial in early training phases to build adequate ST skill competency before progressing toward high-velocity or high-load ST. Neuromuscular adaptations primarily explain ST-induced gains during the early phases of training, particularly in novice female athletes [11]. Accordingly, neuromuscular development during adolescence is closely tied to maturational status, particularly around the period of peak height velocity [PHV], which marks the phase of maximal skeletal growth. Following PHV, a relative decline in muscular power may occur, particularly in females [12]. This decline can affect athletic performance and injury risk. A meta-analysis revealed that young athletes of any age or sex might benefit from ST [12], although a subsequent systematic review suggested that absolute increases in muscle strength are greater during or after PHV than before PHV [13].

Beyond its neuromuscular benefits, ST also induces significant endocrine adaptations that play a crucial role in athletic development. These hormonal adaptations interact with neuromuscular mechanisms underlying strength gains, which has led to the widespread use of hormonal markers for long-term athlete monitoring [14].

Anabolic hormones such as testosterone (T), growth hormone (GH), and insulin-like growth factor-1 (IGF-1) promote muscle hypertrophy, protein synthesis, and neuromuscular efficiency, whereas catabolic hormones like cortisol (C) can inhibit these processes [15]. Thus, the balance between anabolic and catabolic hormonal activity plays a critical role in mediating strength adaptations in response to training stimuli [15].

Moreover, it is important to highlight that hormonal variations linked to the menstrual cycle represent an important biological factor that may modulate training adaptations and performance in female athletes [16]. Estrogen has been proposed to enhance anabolic responses to training, while progesterone may exert catabolic effects [16].

In this context, blood-based biomarkers can provide an objective evaluation of an athlete’s physiological response to training, as well as their overall health, recovery, and metabolic status [14]. The tracking of changes in these biomarkers may contribute to a better understanding of training-induced adaptations in female team-sport athletes, as well as to the early identification of maladaptive responses to excessive or poorly managed training loads [17].

Therefore, maximizing physical performance while supporting physiological and hormonal adaptations is a key challenge in training young female athletes (herein, soccer players). Accordingly, investigating the combined effects of ST on physical performance and hormonal profiles in this population is highly relevant, as it can inform evidence-based strategies to optimize performance, protect athlete health, and promote long-term athletic development during this critical period of maturation.

Although research on hormonal adaptations to ST in youth female athletes is limited, the available studies conducted with youth (e.g., Jansson et al. [18]) have applied heterogeneous ST programming lasting from 6 to 12 weeks, with 2–3 weekly sessions at moderate-to-high training intensities and showed that resistance training induced a significant increase in T, in contrast to endurance training which did not produce a detectable hormonal response. No significant effects of exercise training were observed on GH or IGF-1 [18]. Due to heterogeneity in study designs, limited data collected from female populations (i.e., the gender data gap), and the unjustified extrapolation of findings from male to female youth, no consistent pattern of hormonal responses has yet been identified. To the best of our knowledge, no longitudinal study has examined the effects of ST on growth and developmental markers in young elite female soccer players.

Therefore, this study aimed to investigate the effects of a twelve-week full-body ST program in combination with soccer training on measures of physical fitness including maximal dynamic strength, vertical jump performance, linear sprint and change-of-direction (CoD) speed, soccer-specific performance (Yo-Yo Intermittent Recovery Test Level 1 [YYIRTL1], repeated shuttle sprint ability [RSSA]), and hormonal responses (serum T, C, GH, and IGF-1) in elite youth female soccer players with Tier 4 training and performance caliber. We hypothesized that in-season ST would enhance physical fitness in female soccer players by modulating hormonal balance, particularly through anabolic and catabolic variations [4]. This paper serves as a companion to previously published studies (Darragi et al. [8] and Bousselmi et al. [19]) that involved the same participant sample and employs analogous methodology and procedures. Nonetheless, the principal research objectives diverge between previous studies and the current investigation. While Darragi et al. [8] investigated the impact of a twelve week in-season ST program on physical fitness metrics (e.g., muscle strength, power, linear sprint speed, agility, repeated-sprint ability, aerobic capacity) and injury incidence in adolescent female soccer players, Bousselmi et al. [19] focused on the effects of the same training program on maximal dynamic strength, as well as the fluctuations in muscle damage and inflammatory markers in the same population. The current study is primarily focusing on hormonal adaptations induced by the same twelve week in-season ST regimen thereby providing novel insights into hormonal responses (C, T, GH and IGF-1) to ST in this population.

## Methods

### Participants

This study was derived from the same overall research project of Darragi et al. [8] and Bousselmi et al. [19], but addressed distinct primary outcomes and used a specific analytic sample based on data availability. For transparency, full details are provided in the supplementary file.

An a priori power analysis (G*Power) indicated a minimum sample size of 24 participants, required to achieve a power of 0.90 with an alpha of 0.01 and a small effect size (*f* = 0.43) for the primary outcome (testosterone) [17]. To account for potential attrition, 26 female soccer players from the Tunisian national team were initially recruited.

Participants were randomly assigned to either a ST group (STG, *n* = 13) or an active control group (CG, *n* = 13). All players were classified as Tier 4 athletes (elite/international level) according to McKay et al. [20]. To ensure sample homogeneity, only outfield players with no prior systematic ST experience were included. Goalkeepers were excluded from study participation.

All participants were enrolled in a centralized national development program, attending the same sports high school program, and adhering to a standardized training schedule of five 90-minute soccer sessions and one competitive match per week. Two players sustained injuries and were excluded from the final analysis, resulting in a final sample of 24 players (STG, *n* = 12; CG, *n* = 12; see Table 1 for player characteristics). The study was approved by the institutional ethics committee, and informed consent was obtained from all participants and their parents.

**Table 1:** Characteristics of the study participants at baseline

Groups	Body mass (kg)	Body height (cm)	BMI (kg.m^− 2^)	PHV (years)	Body fat (%)
CG (*n* = 12)	55.6 ± 6.4	163.5 ± 6.5	20.8 ± 1.9	2.0 ± 1.4	26.6 ± 2.6
STG (*n* = 12)	55.6 ± 8.9	164.7 ± 7.3	20.4 ± 3.1	2.2 ± 0.6	25.1 ± 3.2

Data are presented as means ± standard deviations (SD)

*CG *Control group, *STG *Strength-training group, *BMI *Body mass index, *PHV *Peak height velocity

ST was incorporated into this framework and maintained consistently throughout the twelve-week intervention, even during temporary participation in club matches or national team training camps. Participants also attended two one-hour physical education classes per week. Nutritional intake and hydration were managed by the academy’s nutrition staff, who provided guidelines for meals consumed outside the academy, particularly on the weekends. All participants were familiarized with the experimental procedures, and both players and their parents or legal representatives provided written informed consent prior to study participation.

Ethical approval was obtained from the Ethics Committee of the University of Sfax (Comité de Protéction des Personnes Sud, C.P.P.SUD No. 0494/2023, Tunisia) and the Tunisian Soccer Federation Medical Ethics Committee. The study procedures adhered to the latest version of the Declaration of Helsinki. Moreover, our study was retrospectively registered as a clinical trial on March 21st, 2025 (number PACTR202504552619186 https://pactr.samrc.ac.za/Search.aspx).

### Procedures

Athletes were tested during the in-season (competitive period) in November, prior to the training program and in February of the next year after the training program was completed.

**Fig. 1:**
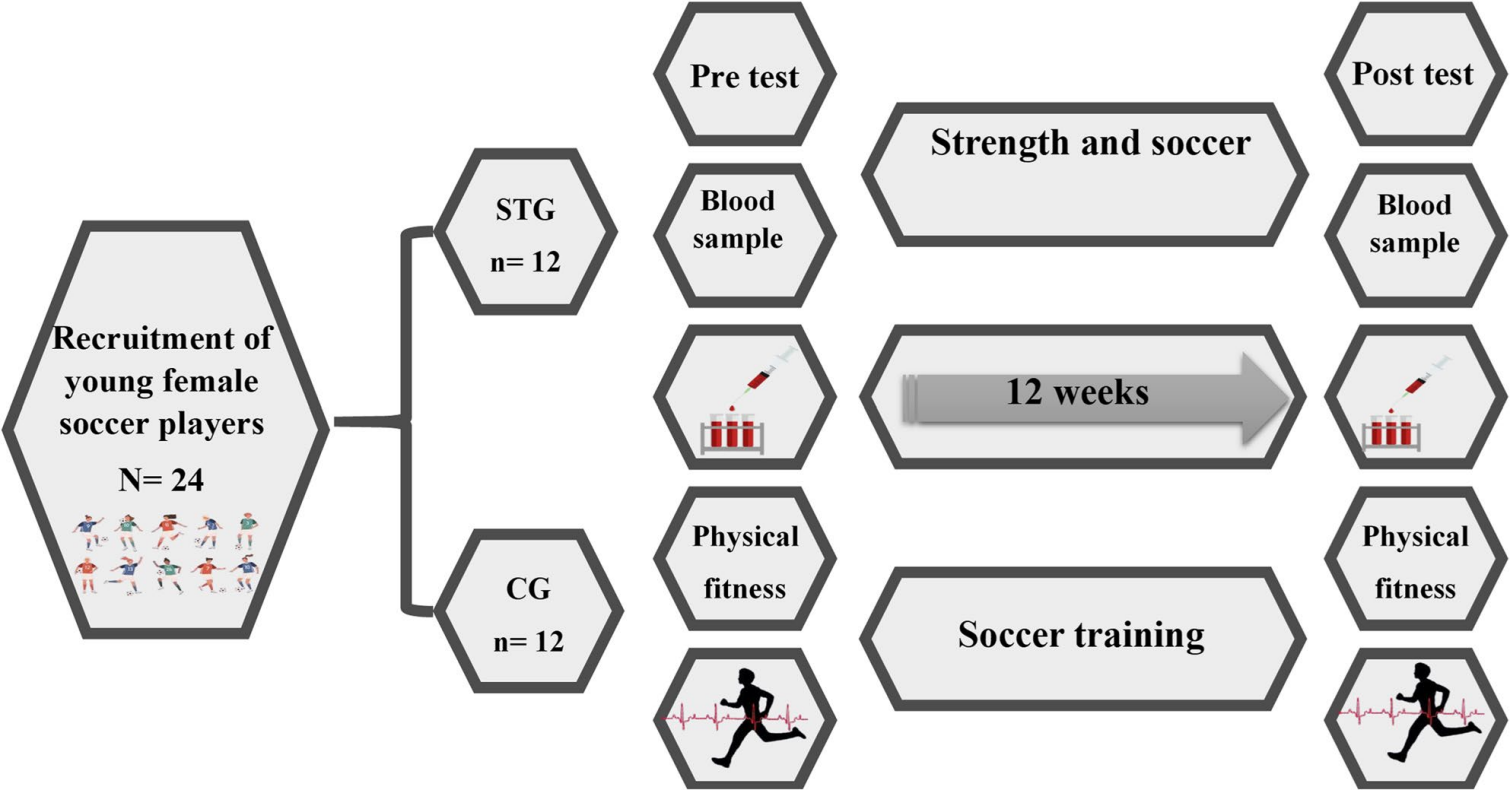
Study design. STG Strength-training group, CG Control group, N Number of participants

Players were tested for their maximal dynamic muscle strength (i.e., relative and absolute one repetition maximum [1-RM] leg/ bench press and lat pull-down), vertical jump performance (i.e., countermovement jump [CMJ]), linear sprint speed (i.e., 5-m 10-m, and 30-m sprint), CoD speed (i.e., T-test with and without ball), and sport-specific performance (i.e., Yo-Yo Intermittent Recovery Test level 1 [YYIRTL1], repeated shuttle sprint ability test [RSSA]) as well as serum T, C, GH and IGF-1 (Fig. 1). Before the physical fitness tests, participants performed a standardized warm-up consisting of ten minutes of general exercises, including submaximal running with CoD, as well as vertical and horizontal submaximal jumps. This was followed by a test-specific warm-up that included two familiarization jumps or runs at submaximal intensities. A test-specific warm-up was performed for each protocol assessment. For the strength tests (1-RM leg / bench press, and lat pull-down), the warm-up consisted of two sets of 6–8 repetitions at approximately 40–60% of the estimated 1-RM, followed by 2–3 repetitions at about 70–80% of the 1-RM. For the jump test (CMJ), participants performed two submaximal practice jumps, whereas for the sprint tests (5, 10, and 30 m), they completed two progressive runs over the corresponding distances. For the CoD test (T-test with and without the ball), one submaximal trial was executed at ~ 70% effort. Finally, for the YYIRTL1 and the RSSA, participants performed two shuttle runs at submaximal intensity. This structured warm-up ensured that all players were adequately prepared and familiarized with the specific demands of each test [8].

All physical fitness test sessions were conducted outdoors on a fourth-generation artificial turf field. Players wore soccer boots for the linear sprint speed tests and the YYIRTL1, whereas indoor sport shoes were used for CMJ assessments during pre- and post-tests. Strength testing was performed in the federation’s gym under the supervision of the same evaluators. All physical fitness assessments took place in the afternoon at 4 pm, while blood sampling was performed at 7:00 am in a fasted state. Participants were instructed to maintain proper hydration and achieve a minimum of eight hours of sleep before evaluation. These methodological procedures were implemented to standardize testing conditions between pre- and post-intervention measurements, including hormonal sampling.

### Training program

The study was designed to contrast the effects of a twelve week in-season ST program in combination with soccer training (competitive period) with those of an active control (soccer-specific training only) (Table 2).

**Table 2:**
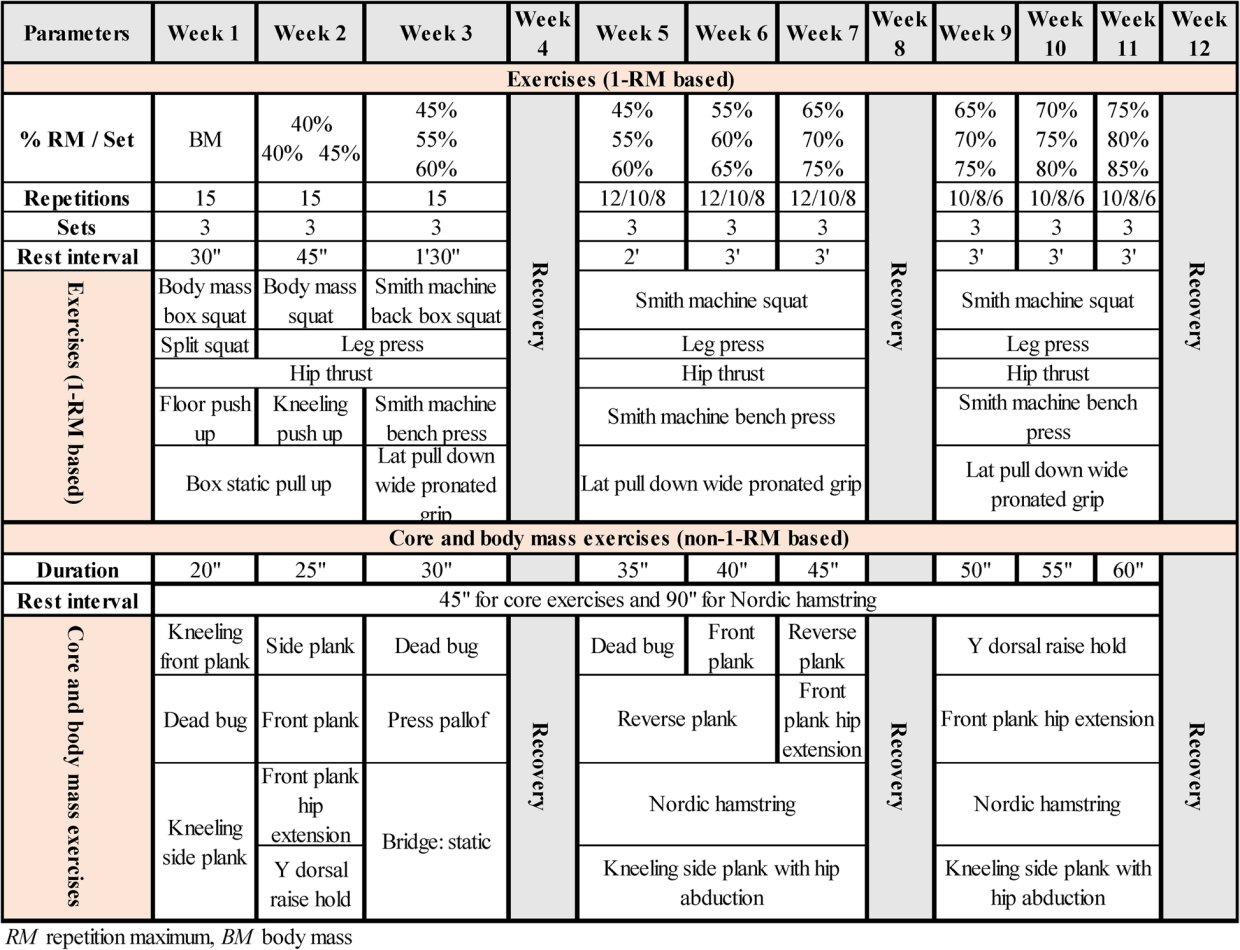
Design of the strength training intervention

The ST program was structured into three four-week cycles, each comprising three weeks of ST (two 90-min sessions per week, separated by at least 48 h) followed by one recovery week with only soccer practice. The program followed a progressive periodization model inspired by Pardos-Mainer et al. [21], with each cycle targeting a specific training objective. Training intensity and exercise selection progressed systematically.

More specifically, body mass exercises were performed first, progressing to machine-based exercises. The 1-RM for machine-based exercises (leg press, lat pull-down, bench press, and smith-machine squat), was tested every four weeks. Training intensity was prescribed as a percentage of the tested 1-RM. The load was progressively increased by 5% per week. Load progression was accompanied by a reduction in the number of repetitions across training cycles, reaching up to 85% of the 1-RM.

Core stabilization exercises were primarily integrated into the warm-up and progressed with regard to exercise difficulty and duration over the training period.

For exercises without measurable 1-RM (e.g., planks, dead bug, Y-hold, and Nordic hamstring), training intensity was controlled by progressively increasing exercise duration, external load, or elastic resistance, depending on the exercise type.

The plank and Y-hold exercises were performed for 3 sets, with the holding duration starting at 15 s and 20 s, respectively, and increased by approximately 5 s per week, reaching up to 55–60 s by week 12.

The dead bug was performed for 3 sets, starting at 6 repetitions per side and increasing by one repetition per week, reaching 14 repetitions per side by week 12.

The Nordic hamstring exercises were performed with external assistance (e.g., partner or elastic support), which was progressively reduced to increase exercise intensity.

Rest intervals were standardized at 45 s for core exercises and 90 s for Nordic hamstring exercises.

To monitor internal training load, the session-RPE was collected approximately 30 min after each training session. Participants were instructed to avoid muscular failure and to maintain proper exercise technique during all sets.

The program included slow, controlled movements, progressive overload based on 1-RM assessments every four weeks, and continuous adherence. The mean attendance rate for ST sessions was 98.3 ± 2.7%, ensuring safe progression and optimal training adaptations.

Each training session began with a warm-up and concluded with a cool-down that included dynamic stretching exercises. A structured warm-up following the RAMP protocol (Raise, Activate, Mobilize, and Potentiate) was performed prior to 1-RM testing. It included four minutes of low-intensity aerobic activity (e.g., treadmill or cycling) to raise body temperature, followed by core and posterior chain activation through body mass exercises such as kneeling plank, side plank, and Y back extension. Joint mobility was enhanced through dynamic movements like thoracic rotations and squat-to-plank. Finally, potentiation was achieved using the first two warm-up sets of the tested exercise.

The exercises incorporated into the ST program have been selected on the basis of their demonstrated efficacy in enhancing strength and power among young athletes. In accordance with the recommendations of Lesinski et al. [9], Pardos-Mainer [21], and Marco-Beato et al. [22], the program placed emphasis on multi-joint movements, including squats, hip thrusts, and Nordic hamstring exercises. Exercises targeting lower limb strength, core stability and strength, as well as movement control, were prioritized, as these are critical components of soccer performance and injury resilience. For the upper body, the program featured the bench press and lat pull-down, which focus on the upper back. Core strength is addressed with the front and side plank exercises.

All ST sessions were supervised by experienced strength and conditioning coaches. The training program was designed to progressively increase training intensity while ensuring proper exercise technique and adequate recovery for the players. Details of weekly exercises, sets, repetitions, and intensity are presented in Table 2.

### Estimation of the menstrual cycle phases

We determined each player’s menstrual cycle phase using the calendar calculation approach [16]. Players were kindly asked to indicate the onset of their menstruation for the three menstrual cycles before the study, from which we computed the average cycle length for each player. We presumed the players to possess a typical ovulatory menstrual cycle if the standard deviation of the cycle length did not surpass three days [16]. The phases of the menstrual cycle were recorded (Tables 3 and 4) and categorized following the methodological guidelines of Jonge et al. [23] into the early follicular phase (days 1 to 4), the late follicular phase (days 10 to 13), and the mid-luteal phase (days 20 to 23), predicated on an average cycle of 28 days [23].

**Table 3:** Phases of the menstrual cycle during the performance of physical fitness tests, pre- and post-training

Physical fitness tests								
Pre					Post			
Groups	EF phase	LF phase	ML phase	Other phases	EF phase	LF phase	ML phase	Other phases
STG	2 (17%)	2 (17%)	2 (17%)	6 (50%)	3 (25%)	3 (25%)	1 (8%)	5 (42%)
CG	4 (33%)	0 (0%)	2 (17%)	6 (50%)	2 (17%)	1 (8%)	4 (33%)	5 (42%)
Total	**6 (25%)**	**2 (8%)**	**4 (17%)**	**12 (50%)**	**5 (21%)**	**4 (17%)**	**5 (21%)**	**10 (42%)**

*CG *Control group, *STG *Strength-training group, *EF *Early follicular, *LF *Late follicular, *ML *Mid-luteal

**Table 4:** Phases of the menstrual cycle during the performance of maximal dynamic strength tests, pre and post training

One-repetition maximum test								
Pre					Post			
Group	EF phase	LF phase	ML phase	Other phases	EF phase	LF phase	ML phase	Other phases
ST	2 (17%)	2 (17%)	2 (17%)	6 (50%)	3 (25%)	1 (33%)	0 (0%)	8 (67%)
CG	0 (0%)	3 (25%)	3 (25%)	6 (50%)	1 (8%)	1 (8%)	4 (33%)	6 (50%)
Total	2 (8%)	5 (21%)	5 (21%)	12 (50%)	4 (17%	2 (8%)	4 (17%	14 (58%)

*CG *Control group, *STG *Strength-training group, *EF *Early follicular, *LF *Late follicular, *ML *Mid-luteal, *1-RM *One-repetition-maximum

### Anthropometric characteristics

Body height and mass were measured using established procedures to the nearest 0.1 cm and 0.1 kg. Body fat was estimated by measuring skin-fold thickness at four sites: triceps, biceps, subscapular, and supra-iliac, utilizing a Harpenden skin-fold caliper (British Indicators Ltd., Luton *UK*) and applying the formula outlined by Durnin and Womersley [24].

Body composition assessments were consistently performed in the morning while fasting, adhering to a uniform technique.

### Maturity status

We measured the maturity status (i.e., years subsequent to peak height velocity) utilizing measurements of both standing and seated height, following the method introduced by Mirwald et al. [25].

$$\begin{aligned}\text{Maturity offset}\;(\mathrm{PHV})\;&=\;-\;9.236\;+\;(0.0002708\;\times\;\text{leg length}\;\times\;\text{sitting height})\;\\&-\;(0.001663\;\times\;\text{chronological age}\;\times\;\text{leg length})\;\\&+\;(0.007216\;\times\;\text{chronological age}\;\times\;\text{sitting height})\;\\&+\;(0.02292\;\times\;(\text{body mass}/\text{body height}\;\times\;100)).\end{aligned}$$

This equation has been previously utilized to determine the maturational status of young female soccer players [26].

### Physical fitness tests

#### Maximal dynamic strength

The maximum dynamic strength evaluation comprised 1-RM tests for the leg/bench press, and lat pull-down, employing weight-lifting machines from Matrix, Houdan, France.

The 1-RM was determined in accordance with the guidelines set forth by the American College of Sports Medicine [27]. A familiarization phase was conducted prior to the official 1-RM testing, during which participants were introduced to the equipment and completed several sets with light loads to familiarize themselves with the testing protocol.

Following a comprehensive dynamic warm-up detailed previously, the assessments began with specific submaximal workouts comprising three lifts with incrementally increasing loads (40%, 75%, and 85% of the anticipated 1-RM). The frequency of repetitions diminished as the session advanced (10→6→3). The initial effort was conducted with a load of 5% under the anticipated 1-RM. Upon a successful lift, the load was augmented by roughly 5%, and the test concluded when the participant was unable to lift the load after 2–3 attempts. The highest load that was successfully lifted was recorded as 1-RM. Participants recuperated for three minutes between lifts [27]. Several studies, including systematic reviews and meta-analyses by Grgic et al. [28], have demonstrated that the 1-RM test shows good to excellent test–retest reliability across a wide range of populations. This high level of reliability appears consistent regardless of participants’ resistance training experience, the number of familiarization sessions, the type of exercise used, the body region tested (upper or lower body), as well as age or sex.

#### Muscle power related tests

As a proxy of muscle power, vertical jump height was tested using the CMJ. The jump height produced during these vertical jumps was quantified with an opto-electric jump system (Opto Jump Microgate, Bolzano, Italy). Three trials were performed, with a 1-minute rest between each, and the highest-performing trial was chosen for further statistical analysis.

The CMJ was utilized to assess maximal jump height performance, incorporating a slow stretch-shortening cycle (SSC) action. The CMJ test employed an optical measurement system, the Optojump (Microgate, Bolzano, Italy), and jump height was determined using a recognized flight-time calculation [12]. While performing the CMJ, participants were directed to position their hands on their hips, adopt a stance with feet and shoulders widely apart, and execute a downward movement succeeded by a maximal vertical jump attempt.

#### Linear sprint speed

Linear sprint speed was recorded using double-light electronic gates (WITTY; Microgate, Bolzano, Italy), positioned at the start line (0 m) and distances of 5, 10, and 30 m, roughly 0.5 m above the ground. Participants commenced the test with one foot placed 15 cm behind the starting line, maintaining an upright posture, and were directed to accelerate as rapidly as possible [9]. They were explicitly instructed to sprint through the final gate without decelerating prematurely. The duration between individual sprint trials varied from 3 to 5 min, and the trial exhibiting the quickest sprint time from two attempts was chosen for statistical analysis.

#### Change-of-Direction speed (CoD)

The T-test was employed as a CoD assessment and conducted by the modified Semenick [29] protocol. Four cones were arranged in a T-shape: cone A (starting point), cone B (10 m straight ahead), and cones C and D (5 m left and right from cone B, respectively). Participants started with both feet behind cone A. At their own discretion, they sprinted forward 10 m to cone B and touched it with the right hand. They then shuffled 5 m to the left to cone C and touched it with the left hand, followed by a 10-m shuffle to the right to cone D and touched it with the right hand. Finally, they shuffled 5 m left back to cone B, touched it with the left hand, and then ran backward to cross the finish line at cone A. Participants executed two attempts, striving for maximal exertion. The trial exhibiting the fastest sprint time of the two was chosen for further data analysis. A 5-minute rest time separated each test trial. The T-test durations (with and without the ball) were documented utilizing the identical photocell technique as in speed tests.

For the “with ball” T-test, players performed the same movement pattern while dribbling a soccer ball with their feet throughout the course. They were instructed to maintain continuous ball control during sprints, shuffles, and backward running, ensuring that both the player and the ball crossed the finish line together.

#### Repeated shuttle sprint ability

After a standardized 15-minute warm-up, players performed six sets of 20-meter sprints (20 m forward and 20 m back) at maximum velocity, with each sprint followed by 20 s of passive rest. Sprint times were documented utilizing the identical photocell technique as in prior experiments, and the optimal time was chosen for the final data analysis (RSSA). Thereafter, the percentage reduction in performance, referred to as RSSA decrement, was computed using the formula: [30].

$$\mathrm{RSSA}\;_\mathrm{Decrement}\;=\;([\text{average RSSA}]\;/\;[\text{improved RSSA}]\;\mathrm{x}\;100)\;-\;100)$$

#### Yo-Yo intermittent recovery test level 1(YYIRTL1)

The Yo-Yo intermittent recovery test level 1 (YYIRTL1) comprises two 20-meter shuttle runs at progressively increasing speeds, regulated by an audio metronome, interspersed with 10-second intervals of active recovery until the participant can no longer sustain the requisite speed at a specific level [31]. Participants were familiarized with the test (performing at least two practice sessions beforehand) and refrained from strenuous exercise 48 h prior. Tests were conducted on the same outdoor artificial turf with the participants wearing soccer shoes. An error threshold of 0.5 m was applied, and standardized verbal cues and encouragement were provided. The test ended when participants failed twice to reach the finishing line in time, with the distance covered recorded as the test result [31].

The recorded score was determined by the overall distance traversed during the final complete shuttle run.

### Training quantification

#### Internal training load

A modified variant of Borg’s session rating of perceived exertion (sRPE) was employed to assess the internal training load (scale from 0 to 10). The sRPE was calculated by multiplying the RPE for the complete training session by the session’s duration. The product of the two factors represented the training load and was expressed in arbitrary units (A.U.) [32]. In this study, raw sRPE values were used as continuous variables.

### Blood samples

Blood samples were taken by a nurse after overnight fasting, and were collected 24 h after the training program at 8.00 am. Blood was collected in a 10 ml tube without additives for each participant. All samples were sent to the Clinical Biochemistry and Hormonology laboratory of the Pasteur Institute “masked for review” on the same day. The transport was done on ice. The tubes were centrifuged 15 min at 3000 rpm and the serum was collected in micro tubes. The serum samples were stored at -20° C for a maximum of two hours after collection and then thawed on the day of the assay. The C and total T were examined using the ECLIA (electro-chemiluminescence immunoassay) technique on the Cobas e411 (Roche Diagnostics, Switzerland) analyzer. The GH and IGF-1 were measured using the radio-immunoassay (RIA) analyzer on the automated STRATEC system (Birkenfeld, Germany) [33].

### Statistical analyses

The statistical analyses were conducted utilizing SPSS software (SPSS, version 26, Chicago, IL) for Windows. The primary sample’s statistics (mean and standard deviation [SD]) were computed for each variable and subsequently classified by group. Prior to inferential analyses, the normality of the data distribution was assessed using the Shapiro-Wilk test, and validity for utilizing parametric procedures was confirmed.

Test reliability was evaluated using inter-session ICC, comparing performance outcomes between pre- and post-test sessions. This inter-session approach was chosen to reflect the stability and reproducibility of the tests across separate days, which is relevant for longitudinal interventions. ICC values were interpreted according to conventional thresholds: <0.50 = poor, 0.50–0.75 = moderate, 0.75–0.90 = good, and > 0.90 = excellent reliability [27]. The coefficient of variation (CV) was calculated to assess reliability, with values ≤ 10% considered indicative of good to acceptable reliability [34].

Intra- and inter-group comparisons were assessed using a two-factor analysis of variance (ANOVA) (2 [groups: STG, CG] × 2 [time: pre, post]). Bonferroni-adjusted post-hoc tests were conducted in the event of significant group-by-time interactions. The F test’s results were contingent upon the significance of Mauchly’s sphericity test. The Greenhouse-Geisser correction was applied if deemed necessary. The effect size was determined by converting partial eta squared from the ANOVA output to Cohen’s d (d) to quantify significant differences in the data. For each effect size, the 95% confidence interval (CI) was also calculated to enhance interpretability. Thresholds for Cohen’s d were defined as trivial (< 0.2), small (0.2–0.59), medium (0.60–1.19), large (1.2–1.99), and very large (≥ 2.0) across all statistical tests, with a significance level set at *p* < 0.05 [34].

## Results

No significant baseline differences between groups were observed for the assessed parameters. All participants fulfilled their training as mandated. Two participants incurred injuries and could not attend all scheduled training sessions, leading to their elimination from the study.

### Anthropometric characteristics

The anthropometric measurements of STG and CG are presented in Table 5. Results showed changes in anthropometric parameters for STG and CG throughout the ST period. A main effect of time was observed for body mass (*p* < 0.001; d = 2.47), BMI (*p* < 0.001; d = 2.39), percentage of body fat (*p* = 0.037; d = 0.95), and lean mass (*p* < 0.001; d = 2.44). Significant group-by-time interactions were found for body mass (*p* = 0.018; d = 1.09) and lean mass (*p* = 0.028; d = 1.01). Post-hoc analyses indicated significant pre-to-post-test increases in lean mass (*p* < 0.01; d = 1.60) and body mass (*p* < 0.01; d = 1.40) within the STG. No significant group-by-time interactions were found for height, body fat percentage, and BMI (0.07 < *p* < 0.19; 0.57 < d < 0.81). Regarding PHV, no significant differences were observed between the two groups, with values of 2.1 ± 0.6 and 2.6 ± 1.0 for STG and CG, respectively.

**Table 5:** Twelve-week strength-training effects on anthropometrics and body composition of female youth Tier 4 soccer players (means ± SDs). Effect sizes are reported as Cohen’s d with 95% confidence intervals (CIs)

Variables	Group	Pre (mean ± SD)	Post (mean ± SD)	% Change	ANOVA p-value (Cohen’s d [95% CI])		
					Main effects		Interaction
					Time	Group	Group x Time
Body fat (%)	STG	25.10 ± 3.25	24.01 ± 2.92	-4.34	0.037* (0.95 [0.1.1; 1.79])	0.080 (0.78 [-0.05; 1.61])	0.193 (0.57 [-0.25; 1.39])
	CG	26.65 ± 2.63	26.38 ± 1.93	-1.01			
Body mass (kg)	STG	55.61 ± 8.88	58.20 ± 9.227	4.66	< 0.001* (2.47 [1.41; 3.53])	0.809 (0.10 [-0.70; 0.90])	0.018* (1.09 [0.23; 1.95])
	CG	55.63 ± 6.39	56.63 ± 6.05	1.80			
Height (cm)	STG	1.64 ± 7.28	1.65 ± 7.31	0.61	0.078 (0.79 [-0.04; 1.62])	< 0.001* (0.07 [-0.73; 0.87])	0.070 (0.81 [-0.02; 1.64])
	CG	1.64 ± 0.06	1.63 ± 0.07	-0.61			
BMI (kg.m-2)	STG	20.36 ± 3.11	21.31 ± 2.76	4.67	< 0.001* (2.39 [1.34; 3.44])	0.824 (0.01 [-0.79; 0.81])	0.111 (0.71 [-0.11; 1.53])
	CG	20.80 ± 1.93	21.32 ± 1.94	2.50			
Lean mass (kg)	STG	43.60 ± 4.94	44.43 ± 5.22	1.90	< 0.001* (2.44 [1.38; 3.50])	0.826 (0.09 [-0.71; 0.89])	0.028* (1.01 [0.16; 1.86])
	CG	43.40 ± 4.51	43.75 ± 4.39	0.81			

*CG *Control group, *STG *Strength-training group, *BMI *Body-mass-index * Significantly different, *p* < 0.05

### Physical fitness tests

#### Reliability

Reliability scores for all physical fitness tests are presented in Table 6. The ICC values indicate moderate to excellent reliability for the different measures taken. More specifically, moderate reliability was found for the CMJ (ICC = 0.65), RSSA _Decrement_ (ICC = 0.58). Good reliability has been indicated by relative and absolute 1-RM bench press with an ICC ranging from 0.86 to 0.88, as well as for relative and absolute 1-RM leg press with an ICC between 0.85 and 0.87. Additionally, the 1-RM lat pull-down showed good reliability, with an ICC of 0.73 for absolute and 0.82 for relative measures, respectively. For 5-m linear sprint speed (ICC = 0.72), the T-test without ball (ICC = 0.86), the YYIRTL1 distance covered (ICC = 0.81), and the 30-m linear sprint test had excellent reliability (ICC = 0.93). Coefficient of variation (CV) values ranged from 1.59% (RSSA _Best_) to 20.98% (absolute 1-RM leg press), reflecting acceptable to high variability depending on the outcome under investigation.

**Table 6:** Relative and absolute reliability of the applied physical fitness tests

Outcome measures	ICC	CV (%)
Relative 1-RM lat pull-down	0.82	12.37
Absolute 1-RM lat pull-down	0.73	14.35
Relative 1-RM bench press	0.88	17.53
Absolute 1-RM bench press	0.86	20.33
Relative 1-RM leg press	0.85	20.98
Absolute 1-RM leg press	0.87	20.40
Countermovement jump	0.65	11.21
5-m sprint	0.72	5.13
10-m sprint	0.86	3.30
30-m sprint	0.93	2.85
RSSA _Decrement_	0.58	4.82
RSSA _Best_	0.72	1.59
YYIRTL1 distance covered	0.81	4.73
T-test	0.85	8.40
T-test _without ball_	0.86	5.22

*ICC *Intraclass correlation coefficient, *CV *Coefficient of variation (%), *1-RM *One repetition maximum, *YYIRTL1 *Yoyo intermittent recovery test level 1, *RSSA *Repeated-shuttle-sprint ability

The baseline physical fitness measures were comparable across STG and CG (Tables 7 and 8). Significant main time effects were observed for the following tests: sprint (5-m, 10-m), CMJ, YYIRTL1, T-test without the ball, RSSA (mean, best), and absolute and relative 1-RM (lat-pull-down, bench press, and leg press) (*p* < 0.05; 0.63 < d < 1.97).

**Table 7:** Twelve-week strength training effects on measures of physical fitness (means ± standard deviations)

Variables	Group	Pre	Post	% Change	ANOVA p-value (Cohen’s d [IC 95%])		
					Main effects		Interaction effect
					Time	Group	Group x Time
CMJ (cm)	STG	23.16 ± 3.23	27.4 ± 3.13	18.26	< 0.001* (1.88 [0.92; 2.84])	0.760 (0.13 [-0.67; 0.93])	0.007* (1.28 [0.40; 2.16])
	CG	24.51 ± 3.14	25.32 ± 3.03	3.30			
Sprint 5-m (s)	STG	1.09 ± 0.05	1.10 ± 0.03	0.92	< 0.001* (1.88 [0.92; 2.84])	0.760 (0.13 [-0.67; 0.93])	0.007* (1.28 [0.40; 2.16])
	CG	1.12 ± 0.09	1.18 ± 0.08	5.36			
Sprint 10-m (s)	STG	1.90 ± 0.07	1.93 ± 0.05	1.58	0.012* (0.79 [-0.04; 1.62])	0.022* (0.72 [-0.11; 1.55])	0.172 (0.42 [-0.39; 1.23])
	CG	1.99 ± 0.12	2.05 ± 0.12	3.02			
Sprint 30-m (s**)**	STG	4.83 ± 0.16	4.77 ± 0.13	-1.24	0.042* (0.63 [-0.19; 1.45])	< 0.001* (1.05 [0.20; 1.90])	0,347 (0.29 [-0.51 ; 1.09])
	CG	5.02 ± 0.29	5.14 ± 0.29	2.39			
T-test with ball (s)	STG	12.24 ± 1.44	10.98 ± 0.93	-10.29	0.236 (0.36 [-0.45; 1.17])	< 0.001* (1.3 [0.42; 2.18])	0.279 (0.33 [-0.48; 1.14])
	CG	13.7 ± 1.04	12.82 ± 0.50	-6.49			
T-test without ball (s)	STG	11.54 ± 0.61	10.72 ± 0.46	-7.11	< 0.001* (1.17 [0.29; 2.05])	< 0.001* (2.15 [1.00; 3.30])	0.913 (0.03 [–0.77; 0.83])
	CG	12.66 ± 0.51	12.06 ± 0.45	-4.74			
YYIRTL1 (m)	STG	891.67 ± 345.62	1303.33 ± 449.0.7	46.17	0.015* (0.76 [–0.09; 1.61])	≤ 0.001* (1.31 [0.39; 2.23])	0.411 (0.25 [–0.57; 1.07])
	CG	611.67 ± 203.33	755.00 ± 329.09	23.43			
RSSA _Total_ (s)	STG	21.50 ± 0.70	21.06 ± 0.59	-2.05	0.203 (0.56 [–0.28; 1.40])	0.002* (1.53 [0.55; 2.51])	< 0.001* (1.85 [0.79; 2.91])
	CG	22.64 ± 1.32	22.88 ± 1.32	1.06			
RSSA _Best_ (s)	STG	3.35 ± 0.16	3.51 ± 0.09	4.78	0.023* (1.04 [0.16; 1.92])	0.280 (0.47 [–0.37; 1.31])	0.070 (0.81 [–0.05; 1.67])
	CG	3.50 ± 0.22	3.5 2 ± 0.27	0.57			
RSSA _Mean_ (s)	STG	3.60 ± 0.15	3.34 ± 0.08	-7.22	< 0.001* (1.97 [0.90; 3.04])	< 0.001* (2.03 [0.93; 3.13])	< 0.001* (2.67 [1.40; 3.94])
	CG	3.77 ± 0.22	3.81 ± 0.21	1.06			
RSSA _Decrement_ (%)	STG	5.05 ± 5.04	2.1 ± 3.3	-58.42	0.244 (0.510 [–0.33; 1.35])	0.336 (0.42 [–0.44; 1.28])	0.369 (0.39[–0.47; 1.25])
	CG	5.89 ± 8.91	5.5 ± 6.5	-6.62			

*CG *Control group, *STG *Strength-training group, *CMJ *Countermovement jump, *YYIRTL1 *Yoyo intermittent recovery test level 1, *RSSA *Repeated-shuttle-sprint ability, *p < 0.05

**Table 8:** Twelve-week strength training effects on maximal dynamic strength (means ± standard deviations)

	Variables	Group	Pre	Post	% Change	ANOVA p-value (Cohen’s d [IC 95%])		
						Main effects		Interaction effect
						Time	Group	Group x Time
Upper limbs	1-RM bench-press (kg)	STG	35.79 ± 4.36	45.2 0 ± 6.00	26.40	< 0.001* (1.38 [0.46; 2.30])	< 0.001* (1.38 [0.46; 2.30])	0.020* (0.70 [–0.16; 1.56])
		CG	35.13 ± 4.67	37.81 ± 4.32	7.63			
	1-RM bench-press relative	STG	0.65 ± 0.06	0.78 ± 0.08	20.00	< 0.001* (1.13 [0.25; 2.01])	< 0.001* (1.37 [0.45; 2.29])	0.044* (0. 60 [–0.24; 1.44])
		CG	0.64 ± 0.14	0.67 ± 0.12	4.69			
Trunk	1-RM lat pull-down (kg)	STG	22.24 ± 3.85	31.15 ± 5.48	40.06	< 0.001* (1.20 [0.30; 2.10])	0.012* (0.75 [–0.11; 1.61])	0.013* (0.74 [–0.12; 1.60])
		CG	19.20 ± 3.92	21.99 ± 3.72	14.53			
	1-RM relative lat pull-down	STG	0.40 ± 0.05	0.54 ± 0.07	35.00	0.013* (0.75 [–0.11; 1.61])	0.024* (0.67 [–0.19; 1.53])	0.090 (0.50 [–0.34; 1.34])
		CG	0.35 ± 0.09	0.39 ± 0.08	11.42			
Lower limbs	1-RM leg-press (kg)	STG	63.81 ± 20.41	99.64 ± 26.60	56.15	< 0.001* (1.39 [0.47; 2.31])	< 0.001* (1.13 [0.25; 2.01])	0.068 (0.54 [–0.30; 1.38])
		CG	49.88 ± 12.36	65.51 ± 11.07	31.34			
	1-RM relative leg-press	STG	1.13 ± 0.23	1.70 ± 0.31	50.44	< 0.001* (1.51 [0.56; 2.46])	< 0.001* (1.32 [0.42; 2.22])	0.046* (0.59 [–0.26; 1.44])
		CG	0.91 ± 0.30	1.17 ± 0.25	28.57			

*CG *Control group, *STG *Strength-training group, *1-RM *One repetition maximum, *p < 0.05

Significant group-by-time interactions were found for CMJ, RSSA (mean, total) performance, absolute 1-RM (bench press, lat pull-down), and relative 1-RM performance (bench press, leg press) (0.001 < *p* < 0.046; 0.59 < d < 2.67).

Subsequent post-hoc analyses revealed significant pre-to-post enhancements for the STG in CMJ (*p* = 0.003, d = 1.11), RSSA _Mean_ (*p* < 0.001, d = 1.63), RSSA _Total_ (*p* = 0.007, d = 0.95) and absolute 1-RM bench press (*p* < 0.001, d = 4.06), absolute 1-RM lat pull-down (*p* < 0.001, d = 2.38), relative 1-RM leg press (*p* < 0.001, d = 2.39) and relative 1-RM bench press (*p* < 0.001, d = 3.65) performances.

Importantly, the internal training load (RPE) was similar between the STG and CG, with mean values of 1205.48 ± 43.3 AU and 1175.07 ± 57.08 AU, respectively. No statistically significant differences were observed between groups regarding total training volume over the twelve week ST period (*p* = 0.156; d = 0.59), indicating that the improvements in physical performance in the STG occurred despite comparable internal training loads.

### Blood responses

The results of the analysis of the hormonal markers are displayed in Table 9. No significant main time effects (0.093 < *p* < 0.825; 0.07 < d < 0.53), main group effects (0.485 < *p* < 0.954; 0.02 < d < 0.21), or group-by-time interactions (0.346 < *p* < 0.940, 0.02 < d < 0.29) were observed for C, T, GH, IGF-1 (0.346 < *p* < 0.94, 0.02 < d < 0.29).

**Table 9:** Twelve-week strength training effects on hormonal markers

Variables	Group	Pre	Post	% Change	ANOVA p-value (Cohen’s d)		
					Main effects		Interaction effect
					Time	Group	Group x Time
C (nmol/l)	STG	351.60 ± 90.8	454.10 ± 125.57	29.15	0.093 (0.53[-0.29, 1.35])	0.750 (0.10[-0.70, 0.90])	0.346 (0.29[-0.51, 1.09])
	CG	389.18 ± 176.6	416.62 ± 106.93	7.05			
T (nmol/l)	STG	1.46 ± 1.82	1.33 ± 1.14	-9.51	0.825 (0.07[-0.73, 0.87])	0.954 (0.02[-0.78, 0.82])	0.940 (0.02[-0.78, 0.82])
	CG	1.15 ± 0.60	1.15 ± 0.42	-0.14			
T /C ratio	STG	4 × 10-3 ± 4 × 10-3	3.1 × 10-3 ± 2.6 × 10-3	-21.99	0.467 (0.22[-0.58, 1.02])	0.729 (0.11[-0.69, 0.91])	0.827 (0.07[-0.73, 0.87])
	CG	3.4 × 10-3 ± 1.9 × 10-3	3 × 10-3 ± 1.7 × 10^-^3	-10.92			
GH (ng/ml)	STG	2.18 ± 1.76	1.99 ± 2.32	-8.93	0.713 (0.12[-0.68, 0.92])	0.859 (0.06[-0.74, 0.86])	0.910 (0.04[-0.76, 0.84])
	CG	2.01 ± 2.09	1.64 ± 2.017	-18.08			
IGF-1 (ng/ml)	STG	281.35 ± 110.39	295.40 ± 81.55	4,99	0.466 (0.22[-0.58, 1.02])	0.485 (0.21[-0.59, 1.01])	0.602 (0.16[-0.64, 0.96])
	CG	347.61 ± 66.27	313.23 ± 58.22	-9.89			

*CG *Control group, *STG *Strength-training group, *C *Cortisol, *T *Total testosterone, *GH *Growth hormone, *IGF-1 *Insulin-like growth factor one

## Discussion

This study investigated the effects of a twelve-week ST program on anthropometric and body composition parameters, physical fitness attributes including strength performance indicators, and hormonal alterations in Tier 4 female adolescent soccer players. The main findings revealed significant group-by-time interactions and / or main effects in anthropometric measures and physical fitness characteristics following the intervention, while no notable changes were observed in blood hormonal markers.

### Anthropometric and body composition characteristics

The 12-week ST program elicited significant body composition adaptations in the examined group of adolescent female soccer players (Tier 4). Participants were all post-PHV, with the ST group averaging 2.1 ± 0.6 years and the control group 2.0 ± 0.7 years beyond PHV, confirming that maturation status was controlled. STG demonstrated a 2.1 kg (3.5%) increase in body mass and a concurrent 2.4 kg (5.2%) increase in lean body mass, while the body fat percentage was maintained. These findings align with previous studies reporting that structured ST interventions lasting between 8 and 12 weeks resulted in lean mass gains of approximately 4–6% with modest changes in total body mass in female team sport athletes [11]. The observed increase in lean mass may be practically relevant, as it could be associated with enhanced force-producing capacity, thereby potentially contributing to athletic performance.

### Physical fitness parameters

The main findings of the study showed significant group-by-time interaction in physical fitness parameters (CMJ, RSSA _Mean_, RSSA _Total_, absolute 1-RM [bench press and lat pull-down], and relative 1-RM [bench press, leg press]). These improvements were consistently observed within STG, which showed trivial to very large effect sizes (0.03 ≤ d ≤ 2.67).

Indeed, the choice of appropriate training methods (periodization, programming) can make a considerable difference in the outcome of an ST process [8, 18].

Maximal dynamic strength assessed through the 1-RM test showed significant group-by-time interactions for upper and lower limb and trunk muscles in favor of STG. More precisely, absolute 1-RM values increased for bench press (*p* = 0.020, d = 0.70) and lat pull-down (*p* = 0.013, d = 0.74) exercises, while relative 1-RM metrics improved for bench press (*p* = 0.044, d = 0. 60) and leg press exercises (*p* = 0.046, d = 0.59). These results indicate that the applied training programs effectively increased maximal dynamic strength. This highlights the practical relevance of the applied ST program for young female elite soccer players.

These findings are consistent with previous research demonstrating that ST elicits substantial gains in maximal strength in young female athletes. Similar magnitudes of improvements have been reported following multi-joint ST interventions in adolescent female soccer players [9], as well as after targeted ST such as Nordic hamstring training [35]. Collectively, findings from these studies support our results and indicate that structured ST constitutes an effective stimulus for improving maximal strength in female athletes.

The observed increases are likely attributable to neural and morphological adaptations, particularly in young athletes [36]. Some 1-RM measures showed only moderate reliability (Table 6), so observed increases may partly reflect measurement variability and should be interpreted cautiously.

Muscle power, measured via CMJ height, showed a significant group-by-time interaction in favor of the STG. Specifically, jump height increased by 18.26%, supporting the effectiveness of ST in improving explosive performance. These results align with prior studies showing that ST is among the most effective methods for enhancing maximal strength and jump performance in soccer [37, 38]. In line with Pardos-Mainer et al. [21], who highlighted those interventions lasting ≥ 8 weeks with at least 16 sessions are most effective, our program was structured to optimize power development. McKinley et al. [38] reported that eight weeks of free-weight ST produced greater CMJ gains than soccer-specific training, while Fischerova et al. [39] observed similar improvements in highly trained female players after six weeks of multi-joint, progressive loading exercises.

Supporting this, Chamera et al. [37] showed that a six-week ST program combined with soccer-specific drills also improved jump performance and lower-limb strength in females.

Previous studies suggested that stronger athletes perform sport-specific ballistic actions more effectively [40], and that maximal squat strength, sprinting, and jumping are closely related in elite players [41]. Although CMJ performance improved, the slow, high-volume ST program was not optimized for power, and moderate CMJ reliability suggests gains should be interpreted cautiously.

Linear sprint (5, 10, and 30 m) and CoD speed (T-test with and without ball) were assessed to evaluate speed-related adaptations. In the present study, no significant group-by-time interactions were observed for linear sprint (5, 10, and 30-m) and CoD performance following the in-season ST program. The observed outcomes may be attributable to the specific design features of our program. These results align with previous findings reporting limited effects of isolated ST on sprint and CoD metrics in adolescent female athletes [21, 26, 42, 43]. For instance, Millar et al. [42] observed no training-induced improvements in linear sprint speed or pro-agility shuttle performance after six weeks of in-season hip thrust or back squat training, despite gains in maximal strength and jump performance. Similarly, Pardos-Mainer et al. [21] reported that single-mode ST without additional sprint- or plyometric-specific drills failed to enhance sprint or CoD performance in adolescent female soccer players. These studies, along with meta-analytic evidence [43], suggest that ST alone, particularly when implemented during the competitive season, may not provide sufficient specificity to induce meaningful gains in high-velocity or multi-directional tasks.

The limited transfer observed in the current study can be explained by the specific neuromuscular qualities required for linear sprint and CoD speed, such as rapid force or torque development [44]. Although participants increased their maximal strength, the heavy-load, slow, and controlled nature of the applied ST program, combined with sets performed near muscular failure, probably inhibited the development of rapid force production qualities, thus limiting improvements in linear sprint and CoD speed. These findings emphasize the value of program specificity when aiming to transfer strength gains to speed-related tasks.

Soccer-specific performance was assessed using the YYIRTL1 and the RSSA test.

Significant main effects of time (d = 0.76) and group (d = 1.31) were observed for YYIRTL1 performance, with pre-to-post-test improvements of 23% in the CG and 46% in the STG. However, no significant group-by-time interaction was found following 12 weeks of ST. These findings are consistent with previous research suggesting that ST, whether loaded or unloaded, does not significantly influence aerobic capacity. Falces et al. [45] observed no decrement in YYIRTL1 performance after a 15-week in-season ST intervention. Although concurrent endurance and ST are often thought to interfere, several studies in young soccer players have shown that RT alone can maintain or even improve aerobic capacity [8, 46]. For instance, Marzouki et al. [47] observed substantial VO₂max increases after RT-only interventions. In our post-PHV female cohort, we found no significant YYIRTL1 gains but, crucially, no loss in high-intensity intermittent endurance. These findings indicate that aerobic performance was largely maintained rather than clearly improved through the ST intervention.

Furthermore, significant main effects of time were noted for RSSA_Mean_ (d = 1.97) and RSSA_Best_ (d = 1.04). Notably, significant group-by-time interactions were identified for RSSA_Mean_ and RSSA_Total_, with reductions of -7.22% and − 2.05%, respectively, favoring the STG. These results align with prior studies demonstrating that ST enhances RSSA performance in young elite soccer players [48]. Furthermore, increases in maximal strength have been positively correlated with improvements in sprinting speed, reactive strength, and resisted sprint performance [49], highlighting their beneficial effects on sprint capabilities across team sport athletes, sprinters, and recreational participants [50]. For instance, increasing force output through maximal strength or improving leg stiffness via reactive strength can significantly contribute to sprint performance [50]. These results corroborate the recent systematic review by Osses-Rivera et al. [51], which found that twice-weekly ST protocols (6–12 weeks) improve best, mean and total RSSA times in elite soccer players, regardless of age or sex. Specifically, ST performed at maximal-power intensities enhances best and mean sprint times, while loads of 80–95% 1-RM confer additional benefits to total RSSA performance.

Moreover, the back squat, a cornerstone exercise in our ST program, may have supported improvements in lower-body force production [13, 44]. These improvements should be interpreted considering the moderate reliability of RSSA measures, which may partially affect the magnitude of the observed changes.

### Hormones

#### GH–IGF-1 axis

IGF-1 and GH play key roles in the regulation of muscle mass and protein synthesis, potentially influencing the anabolic response in females [52]. Nonetheless, our findings revealed no significant alterations in resting GH or IGF-1 concentrations within either the STG or the CG. These findings are consistent with prior research demonstrating that female soccer players preserve stable anabolic biomarkers across training periods [53]. In contrast, evidence exists showing declines in IGF-1 and GH in elite female athletes over a season [17]. Such discrepancies may be related to differences in training modalities, intensity, duration, and participant characteristics [52, 54]. Strength training has been shown to acutely increase GH secretion, especially during high-intensity exercises [55]. Nevertheless, acute reactions do not always result in long-term increases in resting GH levels [56]. The elite status of the participants (Tier 4), reflecting long-term training adaptations and natural hormonal fluctuations during the menstrual cycle, may have influenced our findings [56]. Overall, the current findings suggest that a 12-week ST or soccer-specific training regimen may be insufficient to induce observable variations in resting GH or IGF-1 levels in elite, adolescent female soccer players (Tier 4).

### Testosterone

Testosterone (T) may play a role in helping to maintain performance by reducing protein breakdown and promoting protein synthesis [17]. In this study, T levels remained unchanged, showing no significant changes for either STG or CG.

Our hormonal findings regarding T closely align with the previous systematic analysis and meta-analyses of Jansson et al. [18]. These authors reported that T typically exhibited no change in resting concentrations after prolonged training periods of various types of exercise, including ST in youth and adolescents. In contrast, some studies have reported a significant increase in T levels after 11 weeks of training (ballistic strength and soccer training) in elite soccer players [53].

A variety of methodological factors may account for the lack of significant changes observed in our study. The twelve-week in-season training program may not have provided a sufficient duration or intensity to elicit measurable chronic changes in resting T levels of young female soccer players (Tier 4). Second, participants’ high baseline fitness and hormonal stability could have constrained training responses. Additionally, the frequency of blood sampling was limited, which may have missed transient hormonal fluctuations.

### Cortisol

The results for C revealed no statistically significant effects of time, group, or group-by-time interaction following the 12-week ST intervention. These findings align with previous studies reporting no changes in resting C levels after similar interventions in elite female soccer players [52]. The stable resting C levels may reflect high baseline fitness and well-adapted hypothalamic–pituitary–adrenal axis function rather than a lack of training responsiveness. In some contexts, chronic elevations in resting C are considered markers of maladaptation or insufficient recovery capacity [14]; although this view is not held by all researchers [53].

### Testosterone/Cortisol (T/C) ratio

Another commonly used biomarker of training status is the T/C ratio. In our study, this ratio remained stable following both combined ST and soccer training or soccer training only, suggesting that the adolescent female soccer players might not experience excessive fatigue or stress during training [57].

Despite the absence of hormonal changes, the observed increases in muscular strength we feel can be primarily attributed to neuromuscular adaptations [18, 28, 52].

Overall, a stable T/C ratio suggests that the metabolic and hormonal environment likely supported these neuromuscular improvements without any major disruption, although these data alone do not allow strong conclusions about its practical utility for individual monitoring in this context [18, 58].

### Study limitations

The findings of this study are not generalizable to other cohorts with different training and performance caliber. Study results are always specific to the cohort under investigation. Moreover, a larger sample size could have generated more reliable data and may have improved the validity of the results. In addition, the twelve-week intervention provides a relatively short study period, which may limit the ability to detect long-term hormonal adaptations. Future studies with extended training periods could help clarify the time course of training-induced hormonal responses. Measurement reliability can be considered another study limitation. In fact, some performance tests have demonstrated moderate ICC values and relatively high CVs, indicating limited reproducibility. This may have influenced study outcomes, particularly for explosive or intermittent performance measures. Furthermore, certain factors, such as menstrual cycle-related hormonal fluctuations, sleep quality, and psychological stress, were not systematically controlled in this study, which may have impacted the observed hormonal responses.

Additionally, because participants were inexperienced with ST, all exercises were first performed with the players’ own body mass only and at slow execution velocity to ensure technical mastery and safety before progressively introducing external loads. This progressive and cautious approach was necessary to develop adequate ST skill competency; nevertheless, it may have limited the required stimulus to improve RFD, which could partly explain the absence of significant group-by-time interaction in explosive performance measures. Although external loads were individualized based on each athlete’s 1-RM, and internal load was monitored using sRPE, other variables such as movement velocity, velocity loss, and proximity-to-failure were not recorded. The absence of these measures may have influenced the effective training dose and could partially explain the lack of significant hormonal changes.

These methodological considerations should be taken into account when interpreting the results and highlight the need for future studies with longer intervention periods, more precise load monitoring, and comprehensive control of potential confounding factors.

## Conclusions

This study demonstrates the efficacy of a twelve-week ST program in improving physical fitness metrics, encompassing strength, jump, and sprint performances among young female soccer players with Tier 4 training and performance caliber. The most important outcome is that no significant changes were detected in resting hormonal markers (T, C, GH, and IGF-1) under this specific intervention and context. These findings demonstrate the importance of integrating ST into routine in-season programs to enhance athletic performance.

### Practical recommendations

Adolescent elite female soccer players at a Tier 4 training and performance caliber (elite/international level) can safely conduct a twelve-week in-season full-body ST program, consisting of two supervised sessions per week (~ 90 min each) with at least 48 h of recovery between sessions. Begin with a familiarization phase to ensure proper technique, and then implement a progressive periodized program with loads increasing from 40% to 85% of each player’s 1-RM. To more effectively target linear sprint and CoD speed performances, incorporating additional high-velocity, sprint-specific, or plyometric exercises alongside the ST protocol may be beneficial. Future research should further investigate the reasons that lead to the lack of any observed hormonal adaptations among the young female soccer athletes utilized herein.

## Supplementary Information

Supplementary Material 1.

Supplementary Material 2.

Supplementary Material 3.

## Data Availability

The records that were generated and analyzed during the current study are not publicly available due to confidential information about the participants, however, the corresponding author can provide it upon reasonable request.
