# Supplementary material for: Physical fitness and hormonal responses to strength training in adolescent elite female soccer players
Source: BMC Sports Sci Med Rehabil. 2026 Jul 6;18:310. doi: 10.1186/s13102-026-01855-x (PMC13343932; doi:10.1186/s13102-026-01855-x)
Supplement: Supplementary file 2 — Supplementary Material 2. [file 13102_2026_1855_MOESM2_ESM.pdf]

Disclosure: Research cohort used in the scientific project of Darragi M. and Bousselmi M.

To ensure full transparency regarding publications arising from the same research project, we would like to clarify that the present manuscript in BMC Sports Science, Medicine and Rehabilitation (Darragi et al., 2026, under revision), together with a previously published article in Sports Medicine – Open (Darragi et al., 2024), another article published in Sports (Bousselmi et al., 2026), and a fourth manuscript currently under revision in Sports Medicine – Open (Bousselmi et al., under revision), were derived from the same cohort of elite female youth soccer players. Although these studies originated from the same research project, each article addressed distinct and different research questions and outcomes. Specifically, Darragi et al. (2024) examined the effects of a 12-week in-season strength training program on injury incidence and physical fitness. Bousselmi et al. (2026) investigated the effects of the same intervention on biomarkers of muscle damage and inflammatory responses. The present study focused on hormonal responses and physical fitness adaptations following the above-mentioned strength training program. Bousselmi et al. (under revision) evaluated the effects of 12 weeks of strength training on cognitive function, brain-derived neurotrophic factor (BDNF), and insulin-like growth factor-1 (IGF-1). Accordingly, there is no overlap in the primary research objectives, analyses, or reported outcomes across these manuscripts/publications.

The data set used for the 4 articles is also attached as supplementary materials.

The four articles summarized in the table below originate from the same randomized controlled trial but addressed distinct research objectives. In this trial, players were randomly assigned to either a strength training group (STG) or an active control group (CG). The intervention was conducted over a 12-week period during the competitive soccer season and was identical across all four publications. Specifically, participants in the STG completed two additional strength training sessions per week (90 min per session), alongside their regular soccer training schedule of three sessions per week, with at least 48 hours of recovery between strength training sessions. Participants in the CG continued their standard soccer training program, consisting of five regular soccer training sessions per week. Consequently, the overall training volume was comparable between groups.

**Authors; Year of publication; Journal**	**Objectives**	**Difference in sample sizes across the 4 articles (More details in the excel file)**
Darragi M, Zouhal H, Bousselmi M, Karamti HM, Clark CCT, Laher I, Hackney AC, Granacher U, Zouita, AB. Effects of in-season strength training on physical fitness and injury prevention in North African elite young female soccer players. Sports Med Open. 2024; Sep 2;10(1):94. doi:10.1186/s40798-024-00762-0.<br><br>https://link.springer.com/article/10.1186/s40798-024-00762-0#citeas	Effect of a 12-week in-season strength training on measures of physical fitness and injury occurrence in young elite female soccer players.	Thirty players were initially recruited for the study. Following the exclusion of two players who sustained injuries during the intervention period and were unable to complete the study, the final sample comprised 26 participants, with 13 players in each group.
Bousselmi M, Zouita ABM, Darragi M, Karamti HM, Zouita S, Del Coso J, Hmid AB, Hackney AC, Granacher U, Zouhal H. Twelve weeks of in-season strength training at moderate intensities improve strength and body composition without increasing muscle damage or inflammation in elite young female soccer players. Sports (Basel). 2026 Apr 1;14(4):136. doi:10.3390/sports14040136.<br><br>https://www.mdpi.com/2075-4663/14/4/136	Effect of a 12-week in-season strength training on measures of physical fitness and muscle damage (LDH, CPK) and Inflammation markers (TNF-α, IL-6) in young elite female soccer players.	Of the 30 players initially enrolled, data from only 24 participants were included in the final analysis because biological samples were not available or suitable for the laboratory analyses conducted in this study.<br>In the CG, four players were excluded. One sustained an injury that prevented study completion, one was absent during blood sampling, and blood analyses from two additional players could not be completed

		because the cryotubes were damaged during serum thawing for the TNF-α assays.<br>In the STG, two players were excluded. One due to injury and one because no blood sample was available for analysis.<br>Consequently, the final analytic sample comprised 24 players, with 12 participants in each group.
Darragi M**,** Zouita AB, Bousselmi M, Kerir A, Karamti HM, Saeidi A, Hackney AC, Granacher U, Zouhal H. Physical fitness and hormonal responses to strength training in adolescent elite female soccer players. 2026, Under review in BMC Sports Science, Medicine and Rehabilitation.	Effect of a 12-week in-season strength training on measures of physical fitness and hormonal responses in young elite female soccer players.	Of the 30 players initially enrolled, four were excluded because complete blood measurements were unavailable (one player from the STG and three players from the CG), leaving 26 eligible participants. Subsequently, two additional players (one from each group) sustained injuries that prevented them from completing the intervention and were therefore excluded from the analysis. Consequently, the final analytic sample comprised 24 players, with twelve participants in each group.
Bousselmi M, Zouhal H, Darragi M, Karamti HM, Ben Hmid A, Zamali I, Ben Ahmed M, Krir A, Zouita S, Laher I, Hackney AC, Granacher U, Zouita A. Effects of strength training on cognitive function, brain-derived neurotrophic factor and insulin-like growth factor 1 in highly-trained young female soccer players. 2026, Under review in Sports Med. Open.	Effect of a 12-week in-season strength training on physical fitness, soccer performance, cognitive function, and selected markers of neuroplasticity in young female soccer players.	Of the 30 players initially enrolled, the analysis was restricted to 22 participants, as only these players provided biological samples that were available and of sufficient quality for the laboratory analyses performed in this study.

CG: control group, STG: strength training group, LDH: Lactate dehydrogenase, CPK: Creatine phosphokinase, IL-6: interleukin 6, TNF-α: Tumor necrosis factor alpha, IGF-1: insulin-like growth factor 1.

References

Darragi M, Zouhal H, Bousselmi M, Karamti HM, Clark CCT, Laher I, Hackney AC, Granacher U, Zouita, AB. Effects of in-season strength training on physical fitness and injury prevention in North African elite young female soccer players. Sports Med Open. 2024; Sep 2;10(1):94. doi:10.1186/s40798-024-00762-0.
https://doi.org/10.1186/s40798-024-00762-0

Bousselmi M, Zouita ABM, Darragi M, Karamti HM, Zouita S, Del Coso J, Hmid AB, Hackney AC, Granacher U, Zouhal H. Twelve weeks of in-season strength training at moderate intensities improve strength and body composition without increasing muscle damage or inflammation in elite young female soccer players. Sports (Basel). 2026 Apr 1;14(4):136. doi:10.3390/sports14040136.
https://doi.org/10.3390/sports14040136

Darragi M, Zouita AB, Bousselmi M, Kerir A, Karamti HM, Saeidi A, Hackney AC, Granacher U, Zouhal H. Physical fitness and hormonal responses to strength training in adolescent elite female soccer players. 2026, Under review in BMC Sports Science, Medicine and Rehabilitation.

Bousselmi M, Zouhal H, Darragi M, Karamti HM, Ben Hmid A, Zamali I, Ben Ahmed M, Krir A, Zouita S, Laher I, Hackney AC, Granacher U, Zouita A. Effects of strength training on cognitive function, brain-derived neurotrophic factor and insulin-like growth factor 1 in highly-trained young female soccer players. 2026, Under review in Sports Med. Open.
