# Supplementary material for: Physical fitness and hormonal responses to strength training in adolescent elite female soccer players
Source: BMC Sports Sci Med Rehabil. 2026 Jul 6;18:310. doi: 10.1186/s13102-026-01855-x (PMC13343932; doi:10.1186/s13102-026-01855-x)
Supplement: Supplementary file 3 — Supplementary Material 3. [file 13102_2026_1855_MOESM3_ESM.pdf]

Darragi et al ., 2024: Population			
		Players	date of measurement
Control Group	1	Player 1	17/Nov/22
	2	Player 2	17/Nov/22
	3	Player 3	17/Nov/22
	4	Player 4	17/Nov/22
	5	Player 5	17/Nov/22
	6	Player 6	17/Nov/22
	7	Player 7	24/Nov/21
	8	Player 8	17/Nov/22
	9	Player 9	17/Nov/22
	10	Player 10	17/Nov/22
	11	Player 11	17/Nov/22
	12	Player 12	17/Nov/22
	13	Player 13	17/Nov/22
	14	Player 14	17/Nov/22
	15	Player 15	18/Nov/22
	16	Player 16	17/Nov/22
Strength Training Group	17	Player 1	17/Nov/22
	18	Player 2	17/Nov/22
	19	Player 3	17/Nov/22
	20	Player 4	17/Nov/22
	21	Player 5	17/Nov/22
	22	Player 6	17/Nov/22
	23	Player 7	17/Nov/22
	24	Player 8	17/Nov/22
	25	Player 9	17/Nov/22
	26	Player 10	17/Nov/22
	27	Player 11	17/Nov/22
	28	Player 12	17/Nov/22
	29	Player 13	17/Nov/22
	30	Player 14	18/Nov/22

Date of birth	Age	
25/07/2008	14,3	
28/08/2006	16,2	
20/10/2007	15,1	
15/03/2007	15,7	
24/06/2008	14,4	
21/01/2007	15,8	Injuried
19/01/2008	13,8	
24/04/2008	14,6	
25/02/2006	16,7	
04/02/2006	16,8	
04/03/2007	15,7	
05/09/2007	15,2	
09/09/2006	16,2	Injuried
11/08/2006	16,3	
03/02/2006	16,8	Injuried
14/06/2006	16,4	
22/07/2008	14,3	
17/01/2006	16,8	
12/08/2006	16,3	injuried
21/12/2006	15,9	
22/06/2008	14,4	
18/12/2008	13,9	
27/05/2008	14,5	
07/11/2008	14,0	
08/12/2006	15,9	
25/09/2007	15,1	
16/06/2007	15,4	
26/04/2008	14,6	
18/05/2006	16,5	
10/10/2007	15,1	
Mean	15,4	
ET	0,94895	

Darragi et al ., 2024: Population			
		Name and surname	Date of birth
**Control Group**	**1**	Player 1	25/07/2008
	**2**	Player 2	28/08/2006
	**3**	Player 3	20/10/2007
	**4**	Player 4	15/03/2007
	**5**	Player 5	24/06/2008
	**6**	Player 6	21/01/2007
	**7**	Player 7	19/01/2008
	**8**	Player 8	24/04/2008
	**9**	Player 9	25/02/2006
	**10**	Player 10	04/02/2006
	**11**	Player 11	04/03/2007
	**12**	Player 12	05/09/2007
	**13**	Player 13	09/09/2006
	**14**	Player 14	11/08/2006
	**15**	Player 15	03/02/2006
	**16**	Player 16	14/06/2006
**Strength Training Group**	**17**	Player 1	22/07/2008
	**18**	Player 2	17/01/2006
	**19**	Player 3	12/08/2006
	**20**	Player 4	21/12/2006
	**21**	Player 5	22/06/2008
	**22**	Player 6	18/12/2008
	**23**	Player 7	27/05/2008
	**24**	Player 8	07/11/2008
	**25**	Player 9	08/12/2006
	**26**	Player 10	25/09/2007
	**27**	Player 11	16/06/2007
	**28**	Player 12	26/04/2008
	**29**	Player 13	18/05/2006
	**30**	Player 14	10/10/2007

Age	
14,3	
16,2	
15,1	
15,7	
14,4	
15,8	No TNF-alpha analyses
13,8	
14,6	
16,7	
16,8	
15,7	No TNF-alpha analyses
15,2	
16,2	
16,3	
16,8	injured
16,4	No analyses
14,3	No analyses
16,8	
16,3	Injured
15,9	
14,4	
13,9	
14,5	
14,0	
15,9	
15,1	
15,4	
14,6	
16,5	
15,1	
15,4	
0,95	

Bousselmi et al ., 2026: Population

Name and surname	*Date of birth*	*Age*
Player 1	25/07/2008	14,04
Player 2	28/08/2006	16,03
Player 3	20/10/2007	15,01
Player 4	15/03/2007	15,8
Player 5	24/06/2008	14,05
Player 6	19/01/2008	14,1
Player 7	24/04/2008	14,07
Player 8	25/02/2006	16,09
Player 9	04/02/2006	16,09
Player 10	05/09/2007	15,02
Player 11	09/09/2006	16,02
Player 12	11/08/2006	16,03
Player 1	17/01/2006	16,10
Player 2	21/12/2006	15,11
Player 3	22/06/2008	14,05
Player 4	18/12/2008	13,11
Player 5	27/05/2008	14,06
Player 6	07/11/2008	14,00
Player 7	08/12/2006	15,11
Player 8	25/09/2007	15,02
Player 9	16/06/2007	15,05
Player 10	26/04/2008	14,07
Player 11	18/05/2006	15,06
Player 12	10/10/2007	15,01
		14,9
		0,884

**Player** was eliminated because she was injured / **Player** was absent for the blood tests / **Players** were eliminated because their test samples were missing (When thawing the serum, the cryotubes were damaged during the TNF-α assays)
P.S.: **Player**: No blood analysis , **Player** :injured.

Darragi et al ., 2024: Population

		Name and surname	date of measurement
Control Group	1	Player 1	17/Nov/22
	2	Player 2	17/Nov/22
	3	Player 3	17/Nov/22
	4	Player 4	17/Nov/22
	5	Player 5	17/Nov/22
	6	Player 6	17/Nov/22
	7	Player 7	24/Nov/21
	8	Player 8	17/Nov/22
	9	Player 9	17/Nov/22
	10	Player 10	17/Nov/22
	11	Player 11	17/Nov/22
	12	Player 12	17/Nov/22
	13	Player 13	17/Nov/22
	14	Player 14	17/Nov/22
	15	Player 15	18/Nov/22
	16	Player 16	17/Nov/22
Strength Training Group	17	Player 1	17/Nov/22
	18	Player 2	17/Nov/22
	19	Player 3	17/Nov/22
	20	Player 4	17/Nov/22
	21	Player 5	17/Nov/22
	22	Player 6	17/Nov/22
	23	Player 7	17/Nov/22
	24	Player 8	17/Nov/22
	25	Player 9	17/Nov/22
	26	Player 10	17/Nov/22
	27	Player 11	17/Nov/22
	28	Player 12	17/Nov/22
	29	Player 13	17/Nov/22
	30	Player 14	18/Nov/22

Date of birth	Age		Group	
25/07/2008	14,3		CG	1
28/08/2006	16,2	no Hormonal analyses	CG	3
20/10/2007	15,1		CG	4
15/03/2007	15,7		CG	6
24/06/2008	14,4	no Hormonal analyses	CG	7
21/01/2007	15,8		CG	8
19/01/2008	13,8		CG	10
24/04/2008	14,6		CG	11
25/02/2006	16,7	no Hormonal analyses	CG	12
04/02/2006	16,8		CG	13
04/03/2007	15,7		CG	14
05/09/2007	15,2		CG	16
09/09/2006	16,2		STG	17
11/08/2006	16,3		STG	18
03/02/2006	16,8	Injuried	STG	20
14/06/2006	16,4		STG	21
22/07/2008	14,3		STG	22
17/01/2006	16,8		STG	23
12/08/2006	16,3	Injuried	STG	25
21/12/2006	15,9		STG	26
22/06/2008	14,4		STG	27
18/12/2008	13,9		STG	28
27/05/2008	14,5		STG	29
07/11/2008	14,0	no post Hormonal analyses	STG	30
08/12/2006	15,9			
25/09/2007	15,1			
16/06/2007	15,4			
26/04/2008	14,6			
18/05/2006	16,5			
10/10/2007	15,1			
Mean	15,4			
ET	0,94895			

Darragi et al ., Under Review BMC

Name and surname	date of measurement	Date of birth	Age
Player 1	17/Nov/22	25/07/2008	14,3
Player 2	17/Nov/22	20/10/2007	15,1
Player 3	17/Nov/22	15/03/2007	15,7
Player 4	17/Nov/22	21/01/2007	15,8
Player 5	24/Nov/21	19/01/2008	13,8
Player 6	17/Nov/22	24/04/2008	14,6
Player 7	17/Nov/22	04/02/2006	16,8
Player 8	17/Nov/22	04/03/2007	15,7
Player 9	17/Nov/22	05/09/2007	15,2
Player 10	17/Nov/22	09/09/2006	16,2
Player 11	17/Nov/22	11/08/2006	16,3
Player 12	17/Nov/22	14/06/2006	16,4
Player 1	17/Nov/22	22/07/2008	14,3
Player 2	17/Nov/22	17/01/2006	16,8
Player 3	17/Nov/22	21/12/2006	15,9
Player 4	17/Nov/22	22/06/2008	14,4
Player 5	17/Nov/22	18/12/2008	13,9
Player 6	17/Nov/22	27/05/2008	14,5
Player 7	17/Nov/22	08/12/2006	15,9
Player 8	17/Nov/22	25/09/2007	15,1
Player 9	17/Nov/22	16/06/2007	15,4
Player 10	17/Nov/22	26/04/2008	14,6
Player 11	17/Nov/22	18/05/2006	16,5
Player 12	18/Nov/22	10/10/2007	15,1
		Mean	15,3
		SD	0,889

	Populati
	Name and surname
**Control Group**	Player 1
	Player 2
	Player 3
	Player 4
	Player 5
	Player 6
	Player 7
	Player 8
	Player 9
	Player 10
	Player 11
	Player 12
**Strength Training Group**	Player 1
	Player 2
	Player 3
	Player 4
	Player 5
	Player 6
	Player 7
	Player 8
	Player 9
	Player 10
	Player 11
	Player 12

Bousselmi et al ., Under Review Sports Med -open

on BDNF	
Date of birth	***Age***
25/07/2008	14,04
28/08/2006	16,03
20/10/2007	15,01
15/03/2007	15,8
24/06/2008	14,05
19/01/2008	14,1
24/04/2008	14,07
25/02/2006	16,09
04/02/2006	16,09
05/09/2007	15,02
09/09/2006	16,02
11/08/2006	16,03
17/01/2006	16,10
21/12/2006	15,11
22/06/2008	14,05
18/12/2008	13,11
27/05/2008	14,06
07/11/2008	14,00
08/12/2006	15,11
25/09/2007	15,02
16/06/2007	15,05
26/04/2008	14,07
18/05/2006	15,06
10/10/2007	15,01
	14,9
	0,88444

Population BDNF		
Name and surname	***Date of birth***	***Age***
Player 1	25/07/2008	14,04
Player 2	28/08/2006	16,03
Player 3	20/10/2007	15,01
Player 4	24/06/2008	14,05
Player 5	19/01/2008	14,1
Player 6	24/04/2008	14,07
Player 7	25/02/2006	16,09
Player 8	04/02/2006	16,09
Player 9	05/09/2007	15,02
Player 10	09/09/2006	16,02
Player 11	11/08/2006	16,03
Player 1	17/01/2006	16,10
Player 2	21/12/2006	15,11
Player 3	22/06/2008	14,05
Player 4	18/12/2008	13,11
Player 5	27/05/2008	14,06
Player 6	08/12/2006	15,11
Player 7	25/09/2007	15,02
Player 8	16/06/2007	15,05
Player 9	26/04/2008	14,07
Player 10	18/05/2006	15,06
Player 11	10/10/2007	15,01
		14,9
		0,88292

The cryotubes from T1 were damaged during thawing from the serum of Nouha gharssalah and Chams Jerbi for BDNF analysis
